# Supplementary material for: Tracing the Evolutionary Expansion of a Hyperdiverse Antimicrobial Peptide Gene Family in Mytilus spp.: The MyticalinDB Resource
Source: Genes (Basel). 2025 Jul 12;16(7):816. doi: 10.3390/genes16070816 (PMC12294457; doi:10.3390/genes16070816)
Supplement: Supplementary file 1 [file genes-16-00816-s001.zip › genes-3676100-supplementary.pdf]

Supplementary Materials

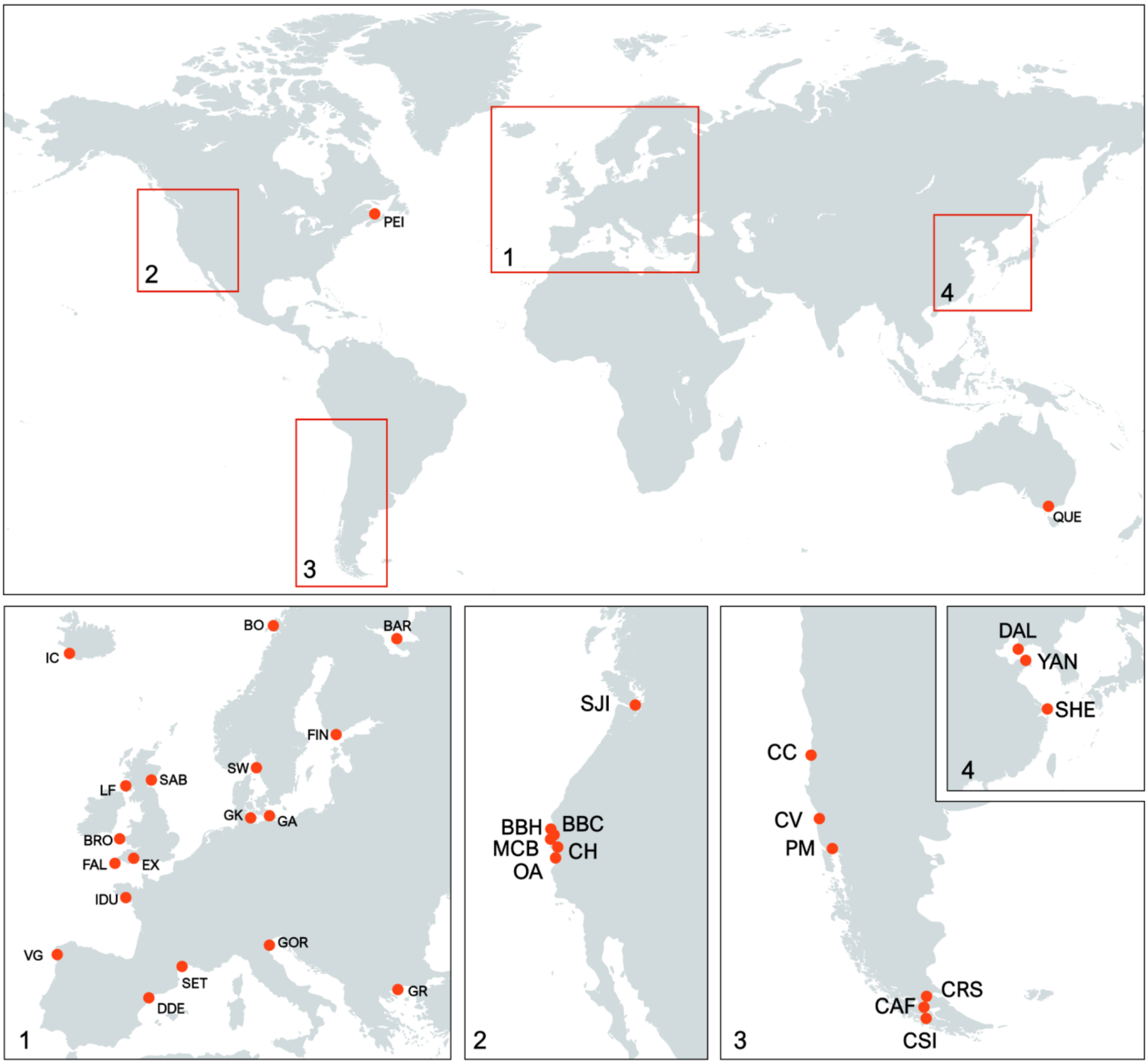

Figure S1. Sampling locations of individuals from the NCBI whose genomes were sequenced and analysed in this study.

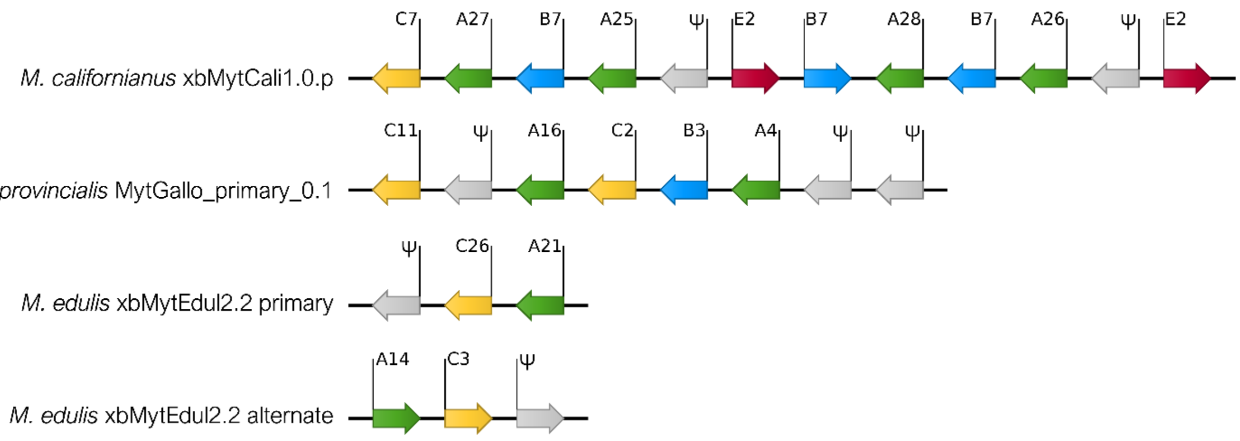

Figure S2. Schematic organization of the myticalin gene clusters detected in four chromosome scale genome assemblies of *Mytilus* spp. ψ indicate pseudogenes.

Table S1. Sampling locations and location codes, associated with genomes obtained from NCBI.

**Location code**	**Full_location**	**Genomes**
DAL	Yellow Sea, Western Pacific Ocean, Dalian, China	DAL1
SJI	San Juan Island, Washington, United States	SJI1
PM	Puerto Montt, SE Pacific Ocean, Chile	PM1; PM151; PM153; PM155; PM161; PM165; PM178
YAN	Yanmaodao, Yellow Sea, China	YAN1
PEI	Prince Edward Island, NE Atlantic Sea, Canada	PEI1
SHE	Shengsi, East China Sea, China	SHE1; SHE2; SHE3
QUE	Queenscliff, Bass Strait, Australia	QUE1
MCB	McClures Beach, NE Pacific Ocean, USA	MCB1
SET	Sete, West Mediterranean Sea, France	SET1
BRO	Broadhaven, Atlantic Ocean, UK	BRO1
SAB	St Andrews Bay, Northern Sea, Scotland	SAB1
BAR	Barents Sea, Most Cherez Kol'skiy Zaliv, Russia	BAR1
IDU	ile Dumet, Vendee, France	IDU1
CAF	Agua Fresca, SE Pacific Ocean, Chile	CAF121; CAF122; CAF123; CAF124; CAF125; CAF126
CRS	Rio Seco, SE Pacific Ocean, Chile	CRS91; CRS92; CRS93; CRS94; CRS95; CRS96
CV	Niebla, SE Pacific Ocean, Chile	CV1; CV2; CV3; CV4; CV5; CV6
DDE	Delta de Ebro, West Mediterranean Sea, Spain	DDE12; DDE13; DDE15; DDE20; DDE27; DDE2
GR	Nea Peramos, Aegean Sea, Greece	GR10; GR14; GR22; GR2; GR4; GR7
SBH	Santa Barbara Harbour, NE Pacific Ocean, USA	SBH1; SBH2; SBH3; SBH4; SBH5; SBH6
OA	Oakland, NE Pacific Ocean, USA	OA1; OA2; OA3; OA4; OA5; OA6
CC	Coliumo, SE Pacific Ocean, Chile	CC1; CC2; CC3; CC4; CC5; CC6
CH	Carquinez Harbour, NE Pacific Ocean, USA	CH1; CH2; CH3; CH4; CH5; CH6
GOR	Goro lagoon, Northern Adriatic Sea, Italy	ITAF1; ITAF2; ITAF3; ITAM1; ITAM2; ITAM3
VG	Ria de Vigo, NE Atlantic Ocean, Spain	PURA; LOLA; GALF1; GALF2; GALF3; GALM1; GALM2; GALM3; GALM6; GALM11; VG12; VG13; VG14; VG15; VG16; VG4
IC	Straumsvik, NE Atlantic Ocean, Iceland	IC1; IC2; IC3; IC4; IC5; IC6

BBC	Bodega Bay Coastal, NE Atlantic Ocean, USA	BBC1; BBC2; BBC3; BBC4; BBC5; BBC6
BBH	Bodega Bay Harbour, NE Pacific Ocean, USA	BBH1; BBH2; BBH3; BBH4; BBH5; BBH6
BO	Bodø, NE Atlantic Ocean, Norway	BO1; BO3; BO6; BO7; BO8; BO9
CSI	San Isidro, SE Pacific Ocean, Chile	CSI61; CSI62; CSI64; CSI65; CSI66; CSI73
EX	River Exe, NE Atlantic Ocean, England	EX1; EX2; EX3; EX4; EX5; EX6
FAL	River Fal, NE Atlantic Ocean, England	FAL25; FAL26; FAL27; FAL30; FAL31; FAL34
FIN	Tvärminne, Baltic Sea, Finland	FIN1; FIN2; FIN3; FIN4; FIN5; FIN6
GA	Ahrenshoop, Baltic Sea, Germany	GA1; GA2; GA3; GA4; GA5; GA6
GK	Kiel, Baltic Sea, Germany	GK1; GK2; GK3; GK4; GK5; GK6
LF	Loch Fyne, NE Atlantic Ocean, Scotland	LF13; LF15; LF171; LF87; LF89; LF93
SW	Kristineberg, NE Atlantic Ocean, Sweden	SW12; SW21; SW24; SW3; SW6; SW7

Table S2. Details of each genome used in this study.

Genome	Reference	Location code	Full_location	Species (*Mytilus)*
DAL1	NCBI WGS master: JAQPZN0000000000.1	DAL	Yellow Sea, Western Pacific Ocean, Dalian, China	*galloprovincialis*
GALF1	Gerdol et al. 2020	VG	Ria de Vigo, NE Atlantic Ocean, Spain	*galloprovincialis*
GALF2	Gerdol et al. 2020	VG	Ria de Vigo, NE Atlantic Ocean, Spain	*galloprovincialis*
GALF3	Gerdol et al. 2020	VG	Ria de Vigo, NE Atlantic Ocean, Spain	*galloprovincialis*
GALM1	Gerdol et al. 2020	VG	Ria de Vigo, NE Atlantic Ocean, Spain	*galloprovincialis*
GALM2	Gerdol et al. 2020	VG	Ria de Vigo, NE Atlantic Ocean, Spain	*galloprovincialis*
GALM3	Gerdol et al. 2020	VG	Ria de Vigo, NE Atlantic Ocean, Spain	*galloprovincialis*
GALM6	Gerdol et al. 2020	VG	Ria de Vigo, NE Atlantic Ocean, Spain	*galloprovincialis*
GALM11	Gerdol et al. 2020	VG	Ria de Vigo, NE Atlantic Ocean, Spain	*galloprovincialis*
ITAF1	Gerdol et al. 2020	GOR	Goro lagoon, Northern Adriatic Sea, Italy	*galloprovincialis*
ITAF2	Gerdol et al. 2020	GOR	Goro lagoon, Northern Adriatic Sea, Italy	*galloprovincialis*
ITAF3	Gerdol et al. 2020	GOR	Goro lagoon, Northern Adriatic Sea, Italy	*galloprovincialis*
ITAM1	Gerdol et al. 2020	GOR	Goro lagoon, Northern Adriatic Sea, Italy	*galloprovincialis*
ITAM2	Gerdol et al. 2020	GOR	Goro lagoon, Northern Adriatic Sea, Italy	*galloprovincialis*
ITAM3	Gerdol et al. 2020	GOR	Goro lagoon, Northern Adriatic Sea, Italy	*galloprovincialis*
ASM167691v1 (PURA)	Murgarella et al. 2016	VG	Ria de Vigo, NE Atlantic Ocean, Spain	*galloprovincialis*
lola_mg3 (LOLA)	Gerdol et al. 2020	VG	Ria de Vigo, NE Atlantic Ocean, Spain	*galloprovincialis*
Mytgalloprovincialis_primary_0.1 + alt	Han et al. 2024	YAN1	Yanmaodao, Yellow Sea, China	*galloprovincialis*
MgalMED	Simon 2022	SET1	Sete, West Mediterranean Sea, France	*galloprovincialis*
MeduEUN	Simon 2022	BAR1	Barents Sea, Most Cherez Kol'skiy Zaliv, Russia	*edulis*
MeduEUS	Simon 2022	IDU1	ile Dumet, Vendee, France	*edulis*
PEIMed_v2	Regan et al. 2024	PEI1	Prince Edward Island, NE Atlantic Sea, Canada	*edulis*
xbMytEdul2.2	NCBI WGS master: CAVMBL000000000	BRO1	Broadhaven, Atlantic Ocean, UK	*edulis*
MEDL1	Corrochano-Fraile et al. 2022	SAB1	St Andrews Bay, Northern Sea, Scotland	*edulis*
xbMytCali1.0.p	Paggeot et al. 2022	MCB1	McClures Beach, NE Pacific Ocean, USA	*californianus*
PNRI_Mtr1.2.1.hap1	NCBI WGS master: JAZBVT000000000.1	SJI1	San Juan Island, Washington, United States	*trossulus*

chilensis reference	Yevenes et al. 2021	PM1	Puerto Montt, SE Pacific Ocean, Chile	*chilensis*
MCOR1.1	Li et al. 2020	SHE1	Shengsi, East China Sea, China	*coruscus*
Mcoruscus_HiC	Yang et al. 2021	SHE2	Shengsi, East China Sea, China	*coruscus*
M. coruscus assembly	Liao et al. 2025: in preparation	SHE3	Shengsi, East China Sea, China	*coruscus*
mussel1.0	NCBI WGS master: APJB000000000	QUE1	Queenscliff, Bass Strait, Australia	*sp*
CAF121	NCBI BioProject: PRJNA932792	CAF	Agua Fresca, SE Pacific Ocean, Chile	*chilensis*
CAF122	NCBI BioProject: PRJNA932792	CAF	Agua Fresca, SE Pacific Ocean, Chile	*chilensis*
CAF123	NCBI BioProject: PRJNA932792	CAF	Agua Fresca, SE Pacific Ocean, Chile	*chilensis*
CAF124	NCBI BioProject: PRJNA932792	CAF	Agua Fresca, SE Pacific Ocean, Chile	*chilensis*
CAF125	NCBI BioProject: PRJNA932792	CAF	Agua Fresca, SE Pacific Ocean, Chile	*chilensis*
CAF126	NCBI BioProject: PRJNA932792	CAF	Agua Fresca, SE Pacific Ocean, Chile	*chilensis*
CRS91	NCBI BioProject: PRJNA932792	CRS	Rio Seco, SE Pacific Ocean, Chile	*chilensis*
CRS92	NCBI BioProject: PRJNA932792	CRS	Rio Seco, SE Pacific Ocean, Chile	*chilensis*
CRS93	NCBI BioProject: PRJNA932792	CRS	Rio Seco, SE Pacific Ocean, Chile	*chilensis*
CRS94	NCBI BioProject: PRJNA932792	CRS	Rio Seco, SE Pacific Ocean, Chile	*chilensis*
CRS95	NCBI BioProject: PRJNA932792	CRS	Rio Seco, SE Pacific Ocean, Chile	*chilensis*
CRS96	NCBI BioProject: PRJNA932792	CRS	Rio Seco, SE Pacific Ocean, Chile	*chilensis*
CV1	NCBI BioProject: PRJNA932792	CV	Niebla, SE Pacific Ocean, Chile	*sp*
CV2	NCBI BioProject: PRJNA932792	CV	Niebla, SE Pacific Ocean, Chile	*sp*
CV3	NCBI BioProject: PRJNA932792	CV	Niebla, SE Pacific Ocean, Chile	*sp*
CV4	NCBI BioProject: PRJNA932792	CV	Niebla, SE Pacific Ocean, Chile	*sp*
CV5	NCBI BioProject: PRJNA932792	CV	Niebla, SE Pacific Ocean, Chile	*sp*
CV6	NCBI BioProject: PRJNA932792	CV	Niebla, SE Pacific Ocean, Chile	*sp*
VG12	NCBI BioProject: PRJNA932792	VG	Ria de Vigo, NE Atlantic Ocean, Spain	*galloprovincialis*
VG13	NCBI BioProject: PRJNA932792	VG	Ria de Vigo, NE Atlantic Ocean, Spain	*galloprovincialis*
VG14	NCBI BioProject: PRJNA932792	VG	Ria de Vigo, NE Atlantic Ocean, Spain	*galloprovincialis*
VG15	NCBI BioProject: PRJNA932792	VG	Ria de Vigo, NE Atlantic Ocean, Spain	*galloprovincialis*
VG16	NCBI BioProject: PRJNA932792	VG	Ria de Vigo, NE Atlantic Ocean, Spain	*galloprovincialis*
VG4	NCBI BioProject: PRJNA932792	VG	Ria de Vigo, NE Atlantic Ocean, Spain	*galloprovincialis*
DDE12	NCBI BioProject: PRJNA932792	DDE	Delta de Ebro, West Mediterranean Sea, Spain	*galloprovincialis*
DDE13	NCBI BioProject: PRJNA932792	DDE	Delta de Ebro, West Mediterranean Sea, Spain	*galloprovincialis*
DDE15	NCBI BioProject: PRJNA932792	DDE	Delta de Ebro, West Mediterranean Sea, Spain	*galloprovincialis*
DDE20	NCBI BioProject: PRJNA932792	DDE	Delta de Ebro, West Mediterranean Sea, Spain	*galloprovincialis*
DDE27	NCBI BioProject: PRJNA932792	DDE	Delta de Ebro, West Mediterranean Sea, Spain	*galloprovincialis*
DDE2	NCBI BioProject: PRJNA932792	DDE	Delta de Ebro, West Mediterranean Sea, Spain	*galloprovincialis*
GR10	NCBI BioProject: PRJNA932792	GR	Nea Peramos, Aegean Sea, Greece	*galloprovincialis*
GR14	NCBI BioProject: PRJNA932792	GR	Nea Peramos, Aegean Sea, Greece	*galloprovincialis*
GR22	NCBI BioProject: PRJNA932792	GR	Nea Peramos, Aegean Sea, Greece	*galloprovincialis*
GR2	NCBI BioProject: PRJNA932792	GR	Nea Peramos, Aegean Sea, Greece	*galloprovincialis*
GR4	NCBI BioProject: PRJNA932792	GR	Nea Peramos, Aegean Sea, Greece	*galloprovincialis*
GR7	NCBI BioProject: PRJNA932792	GR	Nea Peramos, Aegean Sea, Greece	*galloprovincialis*
SBH1	NCBI BioProject: PRJNA932792	SBH	Santa Barbara Harbour, NE Pacific Ocean, USA	*galloprovincialis*
SBH2	NCBI BioProject: PRJNA932792	SBH	Santa Barbara Harbour, NE Pacific Ocean, USA	*galloprovincialis*
SBH3	NCBI BioProject: PRJNA932792	SBH	Santa Barbara Harbour, NE Pacific Ocean, USA	*galloprovincialis*
SBH4	NCBI BioProject: PRJNA932792	SBH	Santa Barbara Harbour, NE Pacific Ocean, USA	*galloprovincialis*

SBH5	NCBI BioProject: PRJNA932792	SBH	Santa Barbara Harbour, NE Pacific Ocean, USA	*galloprovincialis*
SBH6	NCBI BioProject: PRJNA932792	SBH	Santa Barbara Harbour, NE Pacific Ocean, USA	*galloprovincialis*
OA1	NCBI BioProject: PRJNA932792	OA	Oakland, NE Pacific Ocean, USA	*sp*
OA2	NCBI BioProject: PRJNA932792	OA	Oakland, NE Pacific Ocean, USA	*sp*
OA3	NCBI BioProject: PRJNA932792	OA	Oakland, NE Pacific Ocean, USA	*sp*
OA4	NCBI BioProject: PRJNA932792	OA	Oakland, NE Pacific Ocean, USA	*sp*
OA5	NCBI BioProject: PRJNA932792	OA	Oakland, NE Pacific Ocean, USA	*sp*
OA6	NCBI BioProject: PRJNA932792	OA	Oakland, NE Pacific Ocean, USA	*sp*
CC1	NCBI BioProject: PRJNA932792	CC	Coliumo, SE Pacific Ocean, Chile	*chilensis*
CC2	NCBI BioProject: PRJNA932792	CC	Coliumo, SE Pacific Ocean, Chile	*chilensis*
CC3	NCBI BioProject: PRJNA932792	CC	Coliumo, SE Pacific Ocean, Chile	*chilensis*
CC4	NCBI BioProject: PRJNA932792	CC	Coliumo, SE Pacific Ocean, Chile	*chilensis*
CC5	NCBI BioProject: PRJNA932792	CC	Coliumo, SE Pacific Ocean, Chile	*chilensis*
CC6	NCBI BioProject: PRJNA932792	CC	Coliumo, SE Pacific Ocean, Chile	*chilensis*
BBC1	NCBI BioProject: PRJNA932792	BBC	Bodega Bay Coastal, NE Atlantic Ocean, USA	*sp*
BBC2	NCBI BioProject: PRJNA932792	BBC	Bodega Bay Coastal, NE Atlantic Ocean, USA	*sp*
BBC3	NCBI BioProject: PRJNA932792	BBC	Bodega Bay Coastal, NE Atlantic Ocean, USA	*sp*
BBC4	NCBI BioProject: PRJNA932792	BBC	Bodega Bay Coastal, NE Atlantic Ocean, USA	*sp*
BBC5	NCBI BioProject: PRJNA932792	BBC	Bodega Bay Coastal, NE Atlantic Ocean, USA	*sp*
BBC6	NCBI BioProject: PRJNA932792	BBC	Bodega Bay Coastal, NE Atlantic Ocean, USA	*sp*
BBH1	NCBI BioProject: PRJNA932792	BBH	Bodega Bay Harbour, NE Pacific Ocean, USA	*sp*
BBH2	NCBI BioProject: PRJNA932792	BBH	Bodega Bay Harbour, NE Pacific Ocean, USA	*sp*
BBH3	NCBI BioProject: PRJNA932792	BBH	Bodega Bay Harbour, NE Pacific Ocean, USA	*sp*
BBH4	NCBI BioProject: PRJNA932792	BBH	Bodega Bay Harbour, NE Pacific Ocean, USA	*sp*
BBH5	NCBI BioProject: PRJNA932792	BBH	Bodega Bay Harbour, NE Pacific Ocean, USA	*sp*
BBH6	NCBI BioProject: PRJNA932792	BBH	Bodega Bay Harbour, NE Pacific Ocean, USA	*sp*
BO1	NCBI BioProject: PRJNA932792	BO	Bodø, NE Atlantic Ocean, Norway	*sp*
BO3	NCBI BioProject: PRJNA932792	BO	Bodø, NE Atlantic Ocean, Norway	*sp*
BO6	NCBI BioProject: PRJNA932792	BO	Bodø, NE Atlantic Ocean, Norway	*sp*
BO7	NCBI BioProject: PRJNA932792	BO	Bodø, NE Atlantic Ocean, Norway	*sp*
BO8	NCBI BioProject: PRJNA932792	BO	Bodø, NE Atlantic Ocean, Norway	*sp*
BO9	NCBI BioProject: PRJNA932792	BO	Bodø, NE Atlantic Ocean, Norway	*sp*
CH1	NCBI BioProject: PRJNA932792	CH	Carquinez Harbour, NE Pacific Ocean, USA	*galloprovincialis*
CH2	NCBI BioProject: PRJNA932792	CH	Carquinez Harbour, NE Pacific Ocean, USA	*galloprovincialis*
CH3	NCBI BioProject: PRJNA932792	CH	Carquinez Harbour, NE Pacific Ocean, USA	*chilensis*
CH4	NCBI BioProject: PRJNA932792	CH	Carquinez Harbour, NE Pacific Ocean, USA	*galloprovincialis*
CH5	NCBI BioProject: PRJNA932792	CH	Carquinez Harbour, NE Pacific Ocean, USA	*galloprovincialis*
CH6	NCBI BioProject: PRJNA932792	CH	Carquinez Harbour, NE Pacific Ocean, USA	*galloprovincialis*
CSI61	NCBI BioProject: PRJNA932792	CSI	San Isidro, SE Pacific Ocean, Chile	*chilensis*
CSI62	NCBI BioProject: PRJNA932792	CSI	San Isidro, SE Pacific Ocean, Chile	*chilensis*
CSI64	NCBI BioProject: PRJNA932792	CSI	San Isidro, SE Pacific Ocean, Chile	*chilensis*
CSI65	NCBI BioProject: PRJNA932792	CSI	San Isidro, SE Pacific Ocean, Chile	*chilensis*
CSI66	NCBI BioProject: PRJNA932792	CSI	San Isidro, SE Pacific Ocean, Chile	*chilensis*
CSI73	NCBI BioProject: PRJNA932792	CSI	San Isidro, SE Pacific Ocean, Chile	*chilensis*
EX1	NCBI BioProject: PRJNA932792	EX	River Exe, NE Atlantic Ocean, England	*sp*
EX2	NCBI BioProject: PRJNA932792	EX	River Exe, NE Atlantic Ocean, England	*sp*

EX3	NCBI BioProject: PRJNA932792	EX	River Exe, NE Atlantic Ocean, England	*sp*
EX4	NCBI BioProject: PRJNA932792	EX	River Exe, NE Atlantic Ocean, England	*sp*
EX5	NCBI BioProject: PRJNA932792	EX	River Exe, NE Atlantic Ocean, England	*sp*
EX6	NCBI BioProject: PRJNA932792	EX	River Exe, NE Atlantic Ocean, England	*sp*
FAL25	NCBI BioProject: PRJNA932792	FAL	River Fal, NE Atlantic Ocean, England	*sp*
FAL26	NCBI BioProject: PRJNA932792	FAL	River Fal, NE Atlantic Ocean, England	*sp*
FAL27	NCBI BioProject: PRJNA932792	FAL	River Fal, NE Atlantic Ocean, England	*sp*
FAL30	NCBI BioProject: PRJNA932792	FAL	River Fal, NE Atlantic Ocean, England	*sp*
FAL31	NCBI BioProject: PRJNA932792	FAL	River Fal, NE Atlantic Ocean, England	*sp*
FAL34	NCBI BioProject: PRJNA932792	FAL	River Fal, NE Atlantic Ocean, England	*sp*
FIN1	NCBI BioProject: PRJNA932792	FIN	Tvärminne, Baltic Sea, Finland	*sp*
FIN2	NCBI BioProject: PRJNA932792	FIN	Tvärminne, Baltic Sea, Finland	*sp*
FIN3	NCBI BioProject: PRJNA932792	FIN	Tvärminne, Baltic Sea, Finland	*sp*
FIN4	NCBI BioProject: PRJNA932792	FIN	Tvärminne, Baltic Sea, Finland	*sp*
FIN5	NCBI BioProject: PRJNA932792	FIN	Tvärminne, Baltic Sea, Finland	*sp*
FIN6	NCBI BioProject: PRJNA932792	FIN	Tvärminne, Baltic Sea, Finland	*sp*
GA1	NCBI BioProject: PRJNA932792	GA	Ahrenshoop, Baltic Sea, Germany	*sp*
GA2	NCBI BioProject: PRJNA932792	GA	Ahrenshoop, Baltic Sea, Germany	*sp*
GA3	NCBI BioProject: PRJNA932792	GA	Ahrenshoop, Baltic Sea, Germany	*sp*
GA4	NCBI BioProject: PRJNA932792	GA	Ahrenshoop, Baltic Sea, Germany	*sp*
GA5	NCBI BioProject: PRJNA932792	GA	Ahrenshoop, Baltic Sea, Germany	*sp*
GA6	NCBI BioProject: PRJNA932792	GA	Ahrenshoop, Baltic Sea, Germany	*sp*
GK1	NCBI BioProject: PRJNA932792	GK	Kiel, Baltic Sea, Germany	*sp*
GK2	NCBI BioProject: PRJNA932792	GK	Kiel, Baltic Sea, Germany	*sp*
GK3	NCBI BioProject: PRJNA932792	GK	Kiel, Baltic Sea, Germany	*sp*
GK4	NCBI BioProject: PRJNA932792	GK	Kiel, Baltic Sea, Germany	*sp*
GK5	NCBI BioProject: PRJNA932792	GK	Kiel, Baltic Sea, Germany	*sp*
GK6	NCBI BioProject: PRJNA932792	GK	Kiel, Baltic Sea, Germany	*sp*
IC1	NCBI BioProject: PRJNA932792	IC	Straumsvik, NE Atlantic Ocean, Iceland	*sp*
IC2	NCBI BioProject: PRJNA932792	IC	Straumsvik, NE Atlantic Ocean, Iceland	*sp*
IC3	NCBI BioProject: PRJNA932792	IC	Straumsvik, NE Atlantic Ocean, Iceland	*sp*
IC4	NCBI BioProject: PRJNA932792	IC	Straumsvik, NE Atlantic Ocean, Iceland	*sp*
IC5	NCBI BioProject: PRJNA932792	IC	Straumsvik, NE Atlantic Ocean, Iceland	*sp*
IC6	NCBI BioProject: PRJNA932792	IC	Straumsvik, NE Atlantic Ocean, Iceland	*sp*
LF13	NCBI BioProject: PRJNA932792	LF	Loch Fyne, NE Atlantic Ocean, Scotland	*sp*
LF15	NCBI BioProject: PRJNA932792	LF	Loch Fyne, NE Atlantic Ocean, Scotland	*sp*
LF171	NCBI BioProject: PRJNA932792	LF	Loch Fyne, NE Atlantic Ocean, Scotland	*sp*
LF87	NCBI BioProject: PRJNA932792	LF	Loch Fyne, NE Atlantic Ocean, Scotland	*sp*
LF89	NCBI BioProject: PRJNA932792	LF	Loch Fyne, NE Atlantic Ocean, Scotland	*sp*
LF93	NCBI BioProject: PRJNA932792	LF	Loch Fyne, NE Atlantic Ocean, Scotland	*sp*
PM151	NCBI BioProject: PRJNA932792	PM	Puerto Montt, SE Pacific Ocean, Chile	*chilensis*
PM153	NCBI BioProject: PRJNA932792	PM	Puerto Montt, SE Pacific Ocean, Chile	*chilensis*
PM155	NCBI BioProject: PRJNA932792	PM	Puerto Montt, SE Pacific Ocean, Chile	*chilensis*
PM161	NCBI BioProject: PRJNA932792	PM	Puerto Montt, SE Pacific Ocean, Chile	*chilensis*
PM165	NCBI BioProject: PRJNA932792	PM	Puerto Montt, SE Pacific Ocean, Chile	*chilensis*
PM178	NCBI BioProject: PRJNA932792	PM	Puerto Montt, SE Pacific Ocean, Chile	*chilensis*

SW12	NCBI BioProject: PRJNA932792	SW	Kristineberg, NE Atlantic Ocean, Sweden	*edulis*
SW21	NCBI BioProject: PRJNA932792	SW	Kristineberg, NE Atlantic Ocean, Sweden	*edulis*
SW24	NCBI BioProject: PRJNA932792	SW	Kristineberg, NE Atlantic Ocean, Sweden	*edulis*
SW3	NCBI BioProject: PRJNA932792	SW	Kristineberg, NE Atlantic Ocean, Sweden	*edulis*
SW6	NCBI BioProject: PRJNA932792	SW	Kristineberg, NE Atlantic Ocean, Sweden	*edulis*
SW7	NCBI BioProject: PRJNA932792	SW	Kristineberg, NE Atlantic Ocean, Sweden	*edulis*

References

1. Gerdol, M.; Moreira, R.; Cruz, F.; Gómez-Garrido, J.; Vlasova, A.; Rosani, U.; Venier, P.; Naranjo-Ortiz, M.A.; Murgarella, M.; Greco, S.; et al. Massive Gene Presence-Absence Variation Shapes an Open Pan-Genome in the Mediterranean Mussel. *Genome Biol.* **2020**, *21*, 275, doi:10.1186/s13059-020-02180-3.
2. Murgarella, M.; Puiu, D.; Novoa, B.; Figueras, A.; Posada, D.; Canchaya, C. A First Insight into the Genome of the Filter-Feeder Mussel *Mytilus galloprovincialis*. *PLOS ONE* **2016**, *11*, e0151561, doi:10.1371/journal.pone.0151561.
3. Han, G.-D.; Ma, D.-D.; Du, L.-N.; Zhao, Z.-J. Chromosomal-Scale Genome Assembly of the Mediterranean Mussel *Mytilus galloprovincialis*. *Sci. Data* **2024**, *11*, 644, doi:10.1038/s41597-024-03497-5.
4. Simon, A. Three New Genome Assemblies of Blue Mussel Lineages: North and South European *Mytilus edulis* and Mediterranean *Mytilus galloprovincialis* 2022.
5. Regan, T.; Hori, T.S.; Bean, T.P. A Chromosome-Scale *Mytilus edulis* Genome Assembly for Aquaculture, Marine Ecology, and Evolution. *G3 GenesGenomesGenetics* **2024**, *14*, jkae138, doi:10.1093/g3journal/jkae138.
6. Corrochano-Fraile, A.; Davie, A.; Carboni, S.; Bekaert, M. Evidence of Multiple Genome Duplication Events in Mytilus Evolution. *BMC Genomics* **2022**, *23*, 340, doi:10.1186/s12864-022-08575-9.
7. Paggeot, L.X.; DeBiasse, M.B.; Escalona, M.; Fairbairn, C.; Marimuthu, M.P.A.; Nguyen, O.; Sahasrabudhe, R.; Dawson, M.N. Reference Genome for the California Ribbed Mussel, *Mytilus californianus*, an Ecosystem Engineer. *J. Hered.* **2022**, *113*, 681–688, doi:10.1093/jhered/esac041.
8. Gallardo-Escárate, C.; Valenzuela-Muñoz, V.; Nuñez-Acuña, G.; Valenzuela-Miranda, D.; Tapia, F.J.; Yévenes, M.; Gajardo, G.; Toro, J.E.; Oyarzún, P.A.; Arriagada, G.; et al. Chromosome-Level Genome Assembly of the Blue Mussel *Mytilus chilensis* Reveals Molecular Signatures Facing the Marine Environment. *Genes* **2023**, *14*, 876, doi:10.3390/genes14040876.
9. Li, R.; Zhang, W.; Lu, J.; Zhang, Z.; Mu, C.; Song, W.; Migaud, H.; Wang, C.; Bekaert, M. The Whole-Genome Sequencing and Hybrid Assembly of *Mytilus coruscus*. *Front. Genet.* **2020**, *11*, doi:10.3389/fgene.2020.00440.
10. Yang, J.-L.; Feng, D.-D.; Liu, J.; Xu, J.-K.; Chen, K.; Li, Y.-F.; Zhu, Y.-T.; Liang, X.; Lu, Y. Chromosome-Level Genome Assembly of the Hard-Shelled Mussel *Mytilus coruscus*, a Widely Distributed Species from the Temperate Areas of East Asia. *GigaScience* **2021**, *10*, giab024, doi:10.1093/gigascience/giab024.
